# Rehabilitation interventions for depression symptoms among cancer patients in Palestine: A systematic review

**DOI:** 10.3389/fresc.2022.978844

**Published:** 2022-12-05

**Authors:** Maher Mohammad Khalil Battat, Mohammad Marie

**Affiliations:** ^1^ Head Nurse of Bone Marrow Transplant and Leukemia Unit, An-Najah National University Hospital, Nablus, Palestine; ^2^ Faculty of Medicine and Health Sciences, An-Najah National University, Nablus, Palestine

**Keywords:** rehabilitation, intervention, cancer patients, depression symptoms, palliative care, mental health, Palestine

## Abstract

**Background:**

Depression enhances the disease burden in patients with cancer. Psychological interventions and the rehabilitation of depression are required as a part of palliative care for cancer patients to improve their quality of life and mental health.

The aim of the study was to review the literature about depression rehabilitation interventions among patients with cancer in Palestine.

**Method:**

The electronic databases used: PubMed, Science Direct, Research Gate, and Google Scholar to search for systematic review articles for the review study.

**Results:**

A total of 23 articles were reviewed, including five from Palestine and five from Arab and Islamic nations. Pharmacological and non-pharmacological interventions used to decrease symptoms of depression and enhance mental health among cancer patients represent the majority of interventions for depression rehabilitation in cancer patients. Interventions for depression rehabilitation among cancer patients in Palestine are only available from the perspective of palliative care, which also involves family education, managing the symptoms of cancer patients, and providing psychological support.

**Conclusion:**

In Palestine, non-pharmacological interventions, such as psychological interventions, are the primary options for treating and recovering from symptoms of depression. The management of symptoms in cancer patients also has a favorable impact on mental health and recovery from depression. In Palestine, there is a need for improvement in palliative care, particularly interventions for depressive symptoms. The main reason Palestinian patients with cancer have such limited treatment and recovery options are because of Israeli occupation.

## Background

Cancer is the second highest cause of death worldwide (1). In Asia, gastric cancer is the most leading cause of death (2). Similarly, in Palestine, cancer is the second leading cause of death at 14%, exceeded only by heart disease at 30%. Depression has been the most frequently reported mental disorder among patients with cancer that increase disease burden (3). Major depression is characterized as having one or more of the main symptoms of depression (depressed mood and anhedonia) as well as four or more of the other defined symptoms of depression (i.e., weight or appetite change, sleep problems, retardation or psychomotor agitation, exhaustion, lack of control, worthlessness or guilt, decreased concentration, and suicidal ideation over a 2-week period) (4). Globally, the incidence of depression among patients with cancer ranges from 1.5% to over 53% (5). In patients with advanced cancer, depression is associated with a higher symptom burden (6) and, mostly in obese women, affects their quality of life (QoL) (7). Depression has significant associations with gender (female), late stages of cancer (III, IV), and type of cancer (lung cancer, breast cancer, stomach cancer). These associations reflect the need to propose solutions to improve the mental health of cancer patients to increase treatment efficiency (8).

Depression was most common in patients with breast cancer in the Eastern Mediterranean region, and it was twice as common in middle-income countries as it was in low-income countries (9). In Arabic countries, such as Jordan, the prevalence of depression among cancer patients was 51.9% (10), and in Saudi Arabia, the prevalence of depression lasting 1 month was 30.0% among patients with colorectal cancer specifically, which increases the risk for suicidal ideation among depressed patients with cancer (11). In Palestine, the prevalence of depressive symptoms among cancer patients living in the West Bank was 51.9% (12).

However, the Occupied Palestinian territories (OPt) (comprising the West Bank and Gaza Strip) remain under Israeli occupation, prompting many living restrictions, including mental health issues exacerbated by limited access to health care. In addition, there are challenges for healthcare professionals, especially nurses, who need to cross barriers to access healthcare settings, such as Augusta Victoria Hospital and Almakassed Hospital in Jerusalem. Patients also face struggles with Israeli soldiers at checkpoints and barriers (13, 14) as there is a shortage of mental health nurses and services in OPt (15). The Israeli occupation is the main cause of the Sumud culture of response and resilience (16). All these factors contribute to the increased healthcare burden and co-morbid mental illness, leading to anxiety disorders (14), schizophrenia (17), and depression due to a lack of the palliative care required for cancer patients (18). Moreover, patients in the Gaza Strip, in general, and cancer patients, in particular, suffer greatly due to the conditions prevailing in the Strip, from the tight blockade that restricts the freedom of movement (siege closure) of individuals and the requirements of society, and due to the impact of the internal political division. This fact sheet focuses on the reality of cancer patients in the Gaza Strip, and the challenges that prevent them from enjoying their healthcare rights and receiving appropriate health services that take into account the reality of their serious depression and other psychological problems as a result of low socioeconomic status, lack of psychological support in the hospitals, and lack of palliative care (19, 20).

Furthermore, a cross-sectional study in the Palestinian city of Nablus found that cancer patients with chronic pain after breast cancer treatment reported discomfort, anxiety, depression, sleep problems, and poor QoL, indicating that palliative care and social support increase QoL and alleviate pain (21). The pain from procedures was high in pediatric cancer patients with depression (22).

Cancer patients have higher levels of depression than non-cancerous patients (23). Many instruments are used to assess the symptoms of depression in cancer patients, including the Edmonton Symptom Assessment System (ESAS) (6), the Brief Edinburgh Depression Scale (BEDS) (24), and the Hospital Anxiety and Depression Scale (HADS) (25) among others. Integrating early palliative care into the standard oncology care of patients with advanced cancer improves QoL, reduces depression and symptom burden, and leads to survival benefits. The systematic assessment of symptoms must be addressed to overcome the barriers to the implementation of palliative care (26). Palliative care is defined as specific medical services for patients who are experiencing a terminal disease. This method of treatment focuses on relieving the stress and symptoms of the disease from the time of diagnosis and continues through the journey of a serious disease using a holistic approach of palliative rehabilitation. The aim of palliative care is to increase the QoL of the patient and their family (27). This was known method of treatment but the aim of this article was to focus on the concept of depression rehabilitation interventions, which is a significant factor of palliative treatment and recovery for cancer patients and to focus on meeting the mental health needs of cancer patients: interventions to reduce symptoms of depression, and care that enables patients to improve their physical, social, and psychological function while continuing to engage in normal activities (28, 29). Other treatments for depression, such as Omeprazole therapy, elevate 5-HT concentrations in the brain and serum, which influences neuronal activity as well as a variety of other physiological processes, such as cardiovascular function, bowel motility, and platelet aggregation, leading to a reduction in depression (30). Considering the interactions between drugs and geriatric syndromes are common in patients under long-term care (31), while physical exercise at home for the elderly is an effective method of improving QoL (32). Home-based physical activity for the elderly is an effective strategy for reducing the risk of falls and enhancing strength and function.

To the best of our knowledge, there are no studies that clarify the interventions for depression rehabilitation for cancer patients in Palestine. The aim of this study was to review the literature about depression rehabilitation interventions among cancer patients in Palestine.

## Methodology

The articles for this systematic review were attained by searching the PubMed, Science Direct, Research Gate, and Google Scholar databases. The keywords used in the search were as follows: Cancer AND Rehabilitation AND Depression or Mental Health AND intervention OR treatment AND Palestine OR West Bank OR Gaza Strip. Supplementary Appendix A details the search procedure. In addition, a search of Arabic language articles was carried out; full-text articles were translated and critically appraised, redundant articles were omitted, and the articles included in the study were formatted according to the IMRAD style (introduction, method, results, and discussion sections). A quality evaluation checklist was used to evaluate the quality of each study. The checklist included the following items: defined study objectives; a representative sample (with reason); explicit inclusion and exclusion criteria; a valid and reliable assessment of mental health; the rate of response recorded, and losses reported; an acceptable data description; and proper statistical analysis. The quality of the article was examined separately by two investigators (MB and MM), and discrepancies were addressed by the primary investigator (MB). The PICO model used the following: population: cancer patients; intervention: rehabilitation interventions for symptoms of depression; comparison: not applicable; and outcome: mental health and recovery from depression.

A total of 23 studies were reviewed: five from Palestine, five from Arab and Islamic countries, and the remaining 14 publications from international journals on the topic, with 65 references cited. The study was carried out according to the Preferred Reporting Items for Literature Reviews (PRISMA) guidelines, which can be found in Supplementary Appendix B. The characteristics and types of included studies are shown in the matrix in Supplementary Appendix C. The table matrix shows the types of studies that were added: a quasi-experimental design; a secondary analysis of self-reported and clinical measures; a longitudinal mixed-methods study; a descriptive, correlational study; a randomized controlled study; clinical practice guideline; cross-sectional survey; correlational cross-sectional study; cross-sectional and descriptive study; mixed study; longitudinal study; observational cohort hospital-based study; and systematic and literature reviews.

### Inclusion and exclusion criteria

Studies were considered for inclusion if they met the criterion of addressing treatments for depression and interventions for depression rehabilitation for cancer patients.

Old duplicates were excluded.

### Ethics approval and consent to participate

In this study, ethics approval and consent to publish were “Not applicable.”

## Results

Pharmaceutical (such as antidepressants) and non-pharmacological interventions are the two main treatment focuses for depression (e.g., palliative care, family therapy).

### Depression treatments and rehabilitation interventions among cancer patients in other countries

The following studies focused on treatments for depression and rehabilitation interventions among cancer patients worldwide.

Depression affected by activity, inflammation, and self-efficacy was investigated in a quasi-experimental trial for patients who lived more than 10–20 years earlier with advanced cancer after anticancer therapy. The study concluded that exercise, inflammation, and general self-efficacy contribute to changes in depression in a unique way. Cancer patients finished their treatment Cancer patients finished their treatment with a palliative rehabilitation interventions, which included a holistic approach from an interdisciplinary team, leading to a reduction in the systematic inflammatory response in the body as a result of reduced inflammatory markers and body chemistry positively affecting their mental status. Exercise also reduced cancer fatigue and developed self-efficacy (29).

Support groups are situated in recovery centers as well as outpatient services in Germany, according to a review study, and they play an important role in the palliative treatment of cancer patients. These groups are considered as a way for patients to encourage each other to express their feelings of avoidance, denial of problems, feelings of helplessness, and social alienation, all of which are linked to worsened health conditions and QoL. Structured community therapies for cancer survivors reduce depression and improve psychological well-being, QoL, coping, and behavioral adjustment, in addition to improving longevity (33).

In 2017, a secondary analysis of self-reported and clinical measures in Canada, a program of interdisciplinary palliative oncology rehabilitation was designed that helped patients with cancer improve their QoL by enhancing their nutrition, psychological care, and the management of symptoms of depression (34).

A systematic review assumed that the use of pharmacological and non-pharmacological interventions in the treatment of depression among cancer patients was more effective. They reported the antidepressants should be considered in the management of moderate-to-severe major depression in cancer patients, as Mianserin, alprazolam, low-dose fluoxetine, paroxetine, and amitriptyline were shown to be superior at minimizing depressive symptoms, despite the possibility of interference with other existing medications. Moreover, non-pharmacological psychosocial approaches, such as cognitive behavioral counseling, family therapy, palliative care, and the coordination of oncology and mental health providers involved in the treatment of cancer patients through referral to a physician, social work, and other non-pharmacological interventions may also be used (35).

According to a systematic research survey, 45% of reviewed studies found promising results and fewer negative outcomes of herbal complementary therapy compared with conventional medications in reducing depression among cancer patients. Herbal therapies included black cohosh, chamomile, chaste berry, lavender, passionflower, and saffron (36). Another systematic study found that supportive and alternative treatments can help cancer patients with depressive symptoms (37).

A longitudinal mixed-methods study found that supportive treatment paired with rehabilitative and palliative therapies are effective treatments for reducing anxiety and symptoms of depression in cancer patients with high-grade glioma, especially during the postoperative phase (38).

According to a review article published in Japan, more than half of cancer patients experience psychiatric illnesses, such as depression, and they need assistance with the psychological adaptation to cancer pain or recovery. Another function of psycho-oncology is to educate and train hospital and community medical personnel. A psycho-oncologist oversaw the treatment for psychiatric symptoms and medical communication modules of a palliative care educational curriculum. Psychiatrists in cancer treatment environments may help cancer patients and their families in a variety of ways (39).

A WeChat-based education rehabilitation program (WERP) is an important way to alleviate stress and increase QoL in patients with non-small cell lung cancer (NSCLC) after surgical resection, according to a randomized controlled trial. A WERP is a comprehensive health education and rehabilitation program that consists of disease-related health education, rehabilitation exercise guidance, daily activity supervision, and psychological support. It not only assists nurses in learning patients' recovery conditions and detecting mental disorders immediately, allowing them to provide appropriate intervention and guidelines for these patients, but it also assists patients with NSCLC in increasing their understanding of this disease. In addition, we found that a WERP was beneficial for the enhancement of QoL in NSCLC patients, which may be because the WERP helps to not only lessen symptoms of anxiety and depression but also to enhance physical function. As a result, a WERP may enhance the QoL in these NSCLC patients (40).

According to the findings of a quasi-experimental study, counseling would benefit cancer patients by preparing them to overcome stress and psychosocial issues. Emotional distress is considered to be the sixth vital sign to be assessed in cancer patients on a routine basis, along with blood pressure, temperature, pulse, and respiration (41).

A review article concluded that antidepressant treatments are effective and reduce fatigue, pain, and nausea, and improve adherence to cancer treatment. The pretreatment of a cancer patient with antidepressants reduces symptoms of depression induced by interferon alfa. Other non-pharmacological psychosocial interventions can also be applied to reduce depressive symptoms in cancer patients, such as relaxation techniques, individual and group psychotherapy (42), and palliative care counseling (43).

In a quantitative study aimed at examining the prevalence of depressive symptoms and their relationship with QoL domains in-home care patients with cancer in the advanced stages, 86 patients were given psychological tests for depression (Hospital Anxiety Depression [HAD] Scale) and quality of life European Organization for the Research and Treatment of Cancer Quality of Life Questionnaire (EORTC-QLQ-C30) 1 week after admission to the home-care program. The study reported essential methods in treating depression in terminally ill cancer patients by using pharmacological treatment as second- and third-generation tricyclic antidepressants or psych stimulants as well psychological interventions, such as cognitive and behavioral techniques. In patients with advanced cancer who are followed in home care contexts, assessing stress and QoL is critical in order to provide them, their families, and caregivers with appropriate psychosocial therapies (44).

The screening of cancer patients for depression, general management principles (psychology education), pharmacologic or psychological/psychosocial interventions, severity of depression, and a stepped care approach (structured group physical activity programs, group-based peer support, and cognitive behavioral therapy [CBT]) was among the recommended interventions for the treatment of depression among cancer patients in a systematic review (45).

A longitudinal study conducted on cancer patients with bone marrow transplants (BMT) shows the importance of social support in the pre-BMT phase. It predicted depression levels and control for initial levels of depression after a BMT (46).

### Depression rehabilitation interventions and treatments among cancer patients in Arab and Islamic countries

The following studies show depression treatment interventions among cancer patients in Arab and Islamic countries.

A cross-sectional survey was conducted at a major university hospital in Jordan. It showed that depression increased when the patient knew about the cancer and stage of cancer, which indicates the need for more attention to the early screening of depression among cancer patients to provide early interventions such as psychological treatment for depression (10).

Using a 13-item Muslim religiosity scale, a survey of 70 Muslims with colorectal cancer (CRC) in Jeddah, Saudi Arabia, aimed to investigate the prevalence of religious beliefs and traditions in CRC patients and their association with demographic, socioeconomic, psychological, and physical health characteristics. Islamic religious practices, such as community worship and praying (*Fard*) five times a day, reading or reciting the Qur'an several times a week or monthly, *Zakat* (giving money to the needy each year), *Sawm* (fasting during the month of Ramadan and other times), and beliefs in the present, were found to be associated with fewer depressive symptoms and less suicidal ideation in Saudi Arabia (47). In addition, in Saudi Arabia, psychological intervention is indicated to deal with the universal feelings of depression and anxiety about death among cancer patients (48). A cross-sectional and descriptive study was conducted at the Oncology Department at the Military Senior Hospital of Tunis. The study concluded that religious coping strategies are the most effective way of improving QoL and mental health, such as symptoms of depression (49).

An observational cohort hospital-based study in Qatar shows that depression and hopelessness among breast cancer patients decreased when social support increased, by working collaboratively with the multidisciplinary team (50).

### Depression rehabilitation interventions and treatments among cancer patients in Palestine

The following studies will clarify the resources of depression treatment and rehabilitation interventions among cancer patients in Palestine.

The concept of palliative care for cancer patients was officially established by the Al Sadeel Society for Palliative Care in Bethlehem. They started official training for healthcare providers in governmental and non-governmental organizations in Palestine, and provided psychological support for cancer patients with depression as one of their points of care from the perspective of holistic care (51).

A study of 125 breast cancer patients treated by radiation therapy at Augusta Victoria Hospital at Jerusalem concluded that depression is a high burden in women with breast cancer having radiotherapy for a long period of treatment. They reported that some interventions are needed to decrease temporary depression, especially in the early stage of radiotherapy treatment, such as psychosocial support services, including screening routinely for psychosocial issues and providing psychological support from social workers and nurses as needed (25).

Another study from the oncology department at El Nasser Pediatric Hospital in Gaza City found that Palestinian parents of children with cancer had a high rate of mental health issues, with 68% of children with cancer suffering from depression. Parents could gain knowledge and experience by participating in a group conversation, which would be an appropriate time for mothers to draw on their own life stories. This life story outlook offers solid foundations that will help parents feel better and lead to their children's needs being met (22).

A further cross-sectional study conducted at Beit Jala Governmental Hospital in Bethlehem and Augusta Victoria Private Hospital in Jerusalem found that the sons of mothers with late-stage cancer, daughters of mothers with palliative and other treatments (those who had been treated for 3–6 months), and husbands whose wives had radiotherapy treatment (especially those who lived in a village) had higher depression scores. Because of the sons' anxiety regarding predicted death, education of the mothers is an available intervention, and the evaluation of depression among family members is needed, with therapy recommended (52).

In a descriptive and correlational study, 172 patients with lung, breast, colon, prostate, and stomach cancer participated. Depression, fatigue, and quality of life were assessed for patients who were undergoing chemotherapy. Using the conclusion that depression and fatigue were shown to be substantially associated with QoL, healthcare personnel should address the impact of depression when devising or planning treatments to promote QoL for cancer patients through accurate and timely evaluation because fatigue affects the cancer patient's social life and emotional and psychological aspects that affect depression recovery. Based on the assessment of depression symptoms and the start of integrated palliative care, including psychological support to enhance depression rehabilitation and maintain well-being (53).

Finally, clinical studies concluded that palliative treatment is divided into three stages, with treatment being available at all levels of illness, thus allowing the patient to have an acceptable level of control about the depression (54).

The Palestinian studies are summarized in the table below.

**Table:** 

Authors	Available depression treatments
Shawawra and Khleif (51)	Psychological support, such as from palliative care
Shawawra and Khleif(51)	Psychosocial support
Abdelaziz and Mona (22)	Family support by group discussion
Dweib (52)	Psychological educational interventions and palliative care
Dreidi et al. (53)	Family support, psychological support, and exercises “palliative care”

## Discussion

Depression rehabilitation in Palestine is similar in Arabic and Islamic countries, as was found in the review of five studies from Arab and Islamic countries. The studies showed that depression rehabilitation interventions among cancer patients are limited to non-pharmacological interventions of psychosocial and spiritual support (10), Islamic religious coping strategies (47, 49), and hope and social support (50).

Furthermore, 14 studies worldwide used many modalities in the treatment and rehabilitation interventions for depression among cancer patients, which can apply to Palestinian populations. They are classified into two types: pharmacological and non-pharmacological interventions. The pharmacological interventions include antidepressants (43, 45), such as Mianserin, low-dose fluoxetine, paroxetine, and amitriptyline (35), second- and third-generation tricyclic antidepressants (44), and selective serotonin reuptake inhibitors, such as citalopram or escitalopram (45).

The international non-pharmacological interventions used for depression rehabilitation in cancer patients include the following: palliative rehabilitation programs with a holistic approach of care to improve the management of depression in cancer patients that focus on three factors (inflammation, exercise, and self-efficacy) (29); structured group support interventions, such as palliative care in depression rehabilitation clinics (33, 45); programs of interdisciplinary palliative oncology rehabilitation (focusing on nutrition, psychological care, and the management of symptoms of depression in cancer patients as social support) (9, 34); social support (46); other psychological interventions, such as cognitive and behavioral techniques (44); psychosocial interventions (CBT family therapy, palliative care by multidisciplinary team) (21, 34, 35); herbal complementary therapy (chamomile, black cohosh, chasteberry, lavender, passionflower, and saffron) (38) as well as complementary and alternative therapies, such as integrating complementary medicine into the care of childhood cancer survivors with the collaboration of a multidisciplinary team (37, 55); a psycho-oncology approach (education and training program with a management of psychological symptoms module and medical communications module) that supports depressed cancer patients (39); counseling (41); psychosocial interventions as individual and group psychotherapies and relaxation techniques (20, 43); WERPs that are effective in reducing depression among NSCLC patients (40); psychology education, problem-solving techniques, and couples therapy (45); balneotherapy (56); and health resort therapy interventions (57).

The first depression rehabilitation intervention among cancer patients in Palestine, as supported by our literature, is psychological treatment (10, 25) supported by Rodin et al. (35), who mentioned that non-pharmacological interventions can include cognitive behavioral therapy, family therapy, palliative care, and is supported by studies carried out in Jordan (10) and Saudi Arabia (48). The second intervention is patient and family education (53), is found in two international studies (40, 45). Third, the use of palliative care services for the treatment of depression (54), as supported by three studies (38, 40, 45). However, no palliative care was available in Palestine for cancer patients or their families. There was an absence of organizations with strategic planning for palliative care; no presence of educational resources for palliative care; no presence of communication or consultation with the religious man; an absence of bereavement support groups; no active follow-up for the patient and their family; no standards for palliative care services or training programs; no home care service for palliative care; an absence of community awareness for palliative care; and an absence of national standards for palliative care (51). Later, in 2015, the first organization in Palestine to provide palliative care services was the Al-Sadeel Society in Bethlehem, who started a palliative care educational program with Augusta Victoria Hospital to take care of cancer patients (58).

The concept of rehabilitation included the management of physical and mental symptoms in cancer patients, including depression, as physical and psychological symptoms affect each other. Psychotherapeutic techniques have been scientifically proven to be effective in many clinical trials for various disorders to improve psychosocial, cognitive, and occupational outcomes; psychosocial management programs should be added to our clinical standard rehabilitation regimens so that patients achieve good physical health as well as good mental health (59).

In Palestine, we need to screen the symptoms and treatment of depression in patients in the early stages of cancer to prevent their progression to severe mental health problems (12). This is supported by the literature on the need for psychological interventions at an early screening for depression (10). In addition, the relatives of cancer patients also need screening, as supported by a Jordanian study that shows the prevalence of depression among relatives of cancer patients was 81.9% (60). The symptoms of cancer patient need to be assessed to establish palliative care, including depression rehabilitation, as a symptom of cancer pain is associated with the development and exacerbation of psychological distress, including depression (61). In addition, the presence of an abdomino-perineal excision of the rectum affect the QoL of patients with rectal cancer in southern Europe as well as those in Arab (Islamic) countries, affecting religious and cultural factors, such as social isolation (62). Similarly in Jeddah, Saudi Arabia, and Tunis, the method of coping using a religious practice reduced depression in cancer patients (48, 49). However, the religious practice was used in Palestine (63) with no evidence that its use reduced depression among cancer patients.

The literature shows that Palestinian cancer patients need rehabilitation interventions and treatment for depression because they suffer from many issues in their daily life. The literature revealed that mental health was affected negatively in OPt in many ways, especially with limited access to healthcare services for Palestinian patients living in the Gaza Strip, West Bank, and East Jerusalem due to the Israeli Separation Wall and road closures, resulting in a low QoL (15, 64). That lead to increase in mental disorders (14) as depression is higher among Arabs than Jews, especially in women (65). Despite the struggles facing the Palestinian population, they have a coping threshold of resistance and resilience based on the Sumud Islamic culture that assists them in adapting to living with hope and obtaining their freedom (16, 66, 67).

In summary, depression rehabilitation interventions among cancer patients in Palestine are limited to palliative care, including psychosocial support, the management of symptoms, and education.

### Limitations

We first searched for depression rehabilitation among cancer patients, and only a few studies were found regarding the concept of mental health rehabilitation, which led us to search for related depression treatments and interventions among cancer patients to connect them to the available resources in Palestine, as well as to the Arab and Islamic countries. The applicable international strategies used for the recovery for depression in cancer patients are recommended to the local area.

Palestinian cancer patients still face struggles regarding their healthcare rights, which affects their mental health.

### Recommendations

First, we recommend ending the occupation to facilitate the treatment and rehabilitation of cancer patients in Palestine. We can then support, fund, and apply the international rehabilitation strategies for depression among cancer patients in Palestine, including the West Bank and Gaza Strip.

On the other hand, further research is recommended related to depression rehabilitation in Palestine and the use of Islamic religious coping strategies.

## Conclusion

Depression rehabilitation approach essential in the recovery of cancer patient and for cancer survivors to improve and maintain their QoL and mental health. Worldwide, pharmacological and many non-pharmacological interventions are combined for depression rehabilitation among cancer patients. Based on our review of five studies from the Gaza Strip and West Bank, depression rehabilitation interventions among cancer patients in Palestine are mostly non-pharmacological interventions used to decrease symptoms of depression, including psychological treatment (10), psychosocial support services (25), palliative care, and a holistic approach of care for cancer patients (54). In addition, the relatives of cancer patients also need to be assessed and managed for symptoms of depression (52). Similarly, the group discussion for children’s mothers to reflect on their life stories leading to them support their children (22), the education of family and patients regarding depression and how to express their feelings to improve their QoL will also be beneficial (53). On the other hand, pharmacological interventions should be used to control the pain and discomfort in cancer patients, such as pain control medications (54) to decrease symptoms of depression, decrease the cancer burden, and improve the QoL.

## Data Availability

The original contributions presented in the study are included in the article/**Supplementary Material**, further inquiries can be directed to the corresponding author/s.
