# Supplementary material for: Rehabilitation interventions for depression symptoms among cancer patients in Palestine: A systematic review
Source: Front Rehabil Sci. 2022 Dec 5;3:978844. doi: 10.3389/fresc.2022.978844 (PMC9760909; doi:10.3389/fresc.2022.978844)
Supplement: Supplementary file 1 [file Table1.docx]

# Appendixes

Appendix A: Search history for the Palestinian literature.

| **Database/Search engine** | **Search #** | **Search terms/ Keywords/ combinations** | **Number of hits** | **Comments on your search or results (for instance comments on how you have combined terms)** |
| --- | --- | --- | --- | --- |
| **PubMed**  [**https://www.ncbi.nlm.nih.gov/pubmed**](https://www.ncbi.nlm.nih.gov/pubmed) |  | mental health rehabilitation | [41,949](https://pubmed.ncbi.nlm.nih.gov/?term=mental+health+rehabilitation&sort=relevance) |  |
|  |  | cancer patients | [1,699,602](https://pubmed.ncbi.nlm.nih.gov/?term=cancer+patients&sort=relevance) |  |
|  |  | Depression | [533,806](https://pubmed.ncbi.nlm.nih.gov/?term=Depression&sort=) |  |
|  |  | palliative care | palliative care |  |
|  | 1. (1+2+3+4) | (((mental health rehabilitation) AND (cancer patients)) AND (Depression)) AND (palliative care) | **29** | 8 |
| **PubMed**  [**https://www.ncbi.nlm.nih.gov/pubmed**](https://www.ncbi.nlm.nih.gov/pubmed) |  | Edmonton symptom assessment system (ESAS) | 502 |  |
|  |  | Beck depression score | 8,853 |  |
|  |  | cancer patient | 1,567,560 |  |
|  |  | palliative care | 61,307 |  |
|  | 1. (9+7+8+9) | (((palliative care AND )) OR (cancer patient AND ) AND (Beck depression score AND ))) AND (Edmonton 1symptom assessment system AND) | 31 | 2 |
| **Science Direct** |  | mental health rehabilitation for depression among cancer patients |  | 1 |
| **Google Scholar** |  | Rehabilitation and treatment of Depression among cancer patients in Palestine. |  | 3 |
| **Google Scholar** |  | depression treatment among cancer patients in Palestine | 19,200 | 4 |
| **Google Scholar** |  | Depression treatment and rehabilitation in cancer patients in Arabic and islamic countries | 6,480 | 5 |
| **Research Gate** |  | Rehabilitation and treatment of Depression among cancer patients in Palestine. |  | 3 |
