# Supplementary material for: Rehabilitation interventions for depression symptoms among cancer patients in Palestine: A systematic review
Source: Front Rehabil Sci. 2022 Dec 5;3:978844. doi: 10.3389/fresc.2022.978844 (PMC9760909; doi:10.3389/fresc.2022.978844)
Supplement: Supplementary file 3 [file Table3.docx]

| **Title of the study** | **Author** | **Year** | **Aim** | **Methods** | **Measurements** | **Results** |
| --- | --- | --- | --- | --- | --- | --- |
| An interdisciplinary palliative rehabilitation intervention bolstering general self-efficacy to attenuate symptoms of depression in patients living with advanced cancer.[An interdisciplinary palliative rehabilitation intervention bolstering general self-efficacy to attenuate symptoms of depression in patients living with advanced cancer - PubMed (nih.gov)](https://pubmed.ncbi.nlm.nih.gov/25953381/) | [A Feldstain](https://pubmed.ncbi.nlm.nih.gov/?term=Feldstain+A&cauthor_id=25953381) , [S Lebel](https://pubmed.ncbi.nlm.nih.gov/?term=Lebel+S&cauthor_id=25953381), [M R Chasen](https://pubmed.ncbi.nlm.nih.gov/?term=Chasen+MR&cauthor_id=25953381) | 2016 | Examined the unique impact of three program factors that have been shown to improve depression: inflammation, exercise, and self-efficacy. | quasi-experimental design | Serum C-reactive protein, 6-min walk test, General Self-efficacy Scale, and Hospital Anxiety and Depression Scale (depression subscale). | A self-efficacy framework may be a helpful ingredient in interdisciplinary intervention to decrease depressive symptomatology. |
| Reported distress in patients living with advanced cancer: changes pre-post interdisciplinary palliative rehabilitation.  - [Reported distress in patients living with advanced cancer: changes pre-post interdisciplinary palliative rehabilitation - PubMed (nih.gov)](https://pubmed.ncbi.nlm.nih.gov/28474241/) | [Andrea Feldstain](https://pubmed.ncbi.nlm.nih.gov/?term=Feldstain+A&cauthor_id=28474241), [Neil MacDonald](https://pubmed.ncbi.nlm.nih.gov/?term=MacDonald+N&cauthor_id=28474241), [Ravi Bhargava](https://pubmed.ncbi.nlm.nih.gov/?term=Bhargava+R&cauthor_id=28474241) , [Martin Chasen](https://pubmed.ncbi.nlm.nih.gov/?term=Chasen+M&cauthor_id=28474241) | 2017 | To explore changes in self-reported distress for patients who completed the Palliative Rehabilitation Program (PRP), from baseline to program completion. | A secondary analysis of self-report and clinical measures | Distress Thermometer and the Problem checklists | A number of endorsed checklist problems significantly decreased, as did overall self-reported distress. Compared to the existing literature that does not show improvements, our finding begins to support that palliative rehabilitation may benefit patient levels of distress by improving function and quality of life. Psychotherapy, anesthesia, and additional intervention for cognitive difficulties may further benefit patients. |
| 1. Support groups for cancer patients   [Support groups for cancer patients \| SpringerLink](https://link.springer.com/article/10.1007%2Fs00520-003-0536-7)  Google Scholar | [Joachim Weis](https://link.springer.com/article/10.1007%2Fs00520-003-0536-7#auth-Joachim-Weis) | 2003 | This paper provides an overview of the basic approaches and application of support groups for cancer patients and discusses the empirical evidence against the background of intervention research | Literature | Electronic data bases | Many empirical studies have provided evidence-based knowledge that structured group interventions for cancer patients improve psychological wellbeing, reduce anxiety and depression, and improve quality of life, coping and mental adjustment. Positive effects on survival have even been reported, but these effects have not yet been proven. |
| Advanced lung disease: Conclusions: The prevalence of anxiety and depression was found relatively important in our study among cancer patients. *Thus, it is necessary to propose solutions to improve the mental health of cancer patients to increase treatment efficiency. Quality of life and role of palliative care.*[Advanced lung disease: quality of life and role of palliative care - PubMed (nih.gov)](https://pubmed.ncbi.nlm.nih.gov/19170219/) | [Christopher R Gilbert](https://pubmed.ncbi.nlm.nih.gov/?term=Gilbert+CR&cauthor_id=19170219) , [Cecilia M Smith](https://pubmed.ncbi.nlm.nih.gov/?term=Smith+CM&cauthor_id=19170219) | 2019 | Assess quality of life and health-related quality of life has demonstrated the dramatic impact that lung disease has on patients. | Literature review | Electronic data bases | Recognition and correction of nocturnal hypoxemia and other sleep disturbances should enhance quality of life in patients with restrictive lung disease; however, there is currently no evidence to support this claim. End-of-life care needs more attention by clinicians in the decision-making and preparatory phase. Physicians need to maintain their focus on quality-of-life issues as medical management shifts from curative therapies to comfort management therapies. Palliative care and hospice appear to be underused in patients with advanced diseases other than cancer. Because the only curative option for some end-stage restrictive lung diseases is lung transplantation, if transplantation is not an option, palliation of symptoms and hospice care may offer patients and families the opportunity to die with dignity and comfort. |
| Health-related quality of life in patients with high-grade gliomas: a quantitative longitudinal study[Health-related quality of life in patients with high-grade gliomas: a quantitative longitudinal study - PubMed (nih.gov)](https://pubmed.ncbi.nlm.nih.gov/26026860/) | [K Piil](https://pubmed.ncbi.nlm.nih.gov/?term=Piil+K&cauthor_id=26026860) , [J Jakobsen](https://pubmed.ncbi.nlm.nih.gov/?term=Jakobsen+J&cauthor_id=26026860) [^3^](https://pubmed.ncbi.nlm.nih.gov/26026860/#affiliation-3), [K B Christensen](https://pubmed.ncbi.nlm.nih.gov/?term=Christensen+KB&cauthor_id=26026860) [^4^](https://pubmed.ncbi.nlm.nih.gov/26026860/#affiliation-4), [M Juhler](https://pubmed.ncbi.nlm.nih.gov/?term=Juhler+M&cauthor_id=26026860) [^5^](https://pubmed.ncbi.nlm.nih.gov/26026860/#affiliation-5), [M Jarden](https://pubmed.ncbi.nlm.nih.gov/?term=Jarden+M&cauthor_id=26026860) | 2015 | The purpose of this study was to explore physical activity levels, prevalence and severity of anxiety and depressive symptoms and health-related quality of life among patients with a highgrade glioma. | a longitudinal mixed methods study | Karnofsky Performance Status (KPS), physical activity, anxiety and depression and health-related quality of life (FACT-Br) | Supportive care combined with rehabilitative and palliative approaches might well be valuable along the trajectory especially during the post-surgery period when anxiety is at its highest peak |
| The role of psycho-oncology in cancer care in Japan[[The role of psycho-oncology in cancer care in Japan] - PubMed (nih.gov)](https://pubmed.ncbi.nlm.nih.gov/21381296/) | [Nobuya Akizuki](https://pubmed.ncbi.nlm.nih.gov/?term=Akizuki+N&cauthor_id=21381296) | 2010 | Psycho-oncology is expected to play an important role in cancer care | Review | Electronic data bases | More than half of referred patient were diagnosed with adjustment disorders, major depression, or normal reactions. These patients required support in connection with the psychological adjustment to cancer distress or rehabilitation. The education and training of hospital staff and community medical staff is another role for psycho-oncology. In one palliative care training program, a psycho-oncologist directed the psychological symptoms management module and medical communications module. Psychiatrists at cancer centers or designated cancer care hospitals are expected to play multiple roles in the care of cancer patients and their families. They are occasionally unable to meet these expectations, however, due to a lack of understanding by hospital administrators, lack of time to spend on activities other than standard psychiatric services, and, in the case of some psychiatrists, a lack of interest in cancer care. It is important to develop a realistic and effective medical model of cancer care that includes psychiatrists. Training psychiatrists in psycho-oncology is also an important issue. |
| 1. Effectiveness of counseling on depression among cancer patients admitted in pravara rural hospital, Loni (Bk)   [(2) (PDF) Effectiveness of counseling on depression among cancer patients admitted in pravara rural hospital, Loni (Bk) (researchgate.net)](https://www.researchgate.net/publication/285255380_Effectiveness_of_counseling_on_depression_among_cancer_patients_admitted_in_pravara_rural_hospital_Loni_Bk) | [G. Vimala](https://www.researchgate.net/scientific-contributions/G-Vimala-2086669580?_sg%5B0%5D=612xnAznHoTyPOEOquowin8UfaKgqKLiSt_bNKhKCRGom9TlF77s3oxZ_sm7AwDaM1T8yAA.nk9YHXVUVkyBVUoIMpZ45anq4ekOCfq9xGy8vLmTWaxhdHnGKKcU147NibgKbez7tnTxslJUoLD6Yxvc1dO9NA&_sg%5B1%5D=JuwzIwkMSI_0D6VhOBiBx93UGZ9Si1sokkgYtea2CxzTHZ1-uH3_m_O4NjLrJggdAFKmuCo.f0WwXNMx3dzSA9rX-14Z5Jq1MrhKsqZQaFV7yPST5L5NMx3fGXtS9A1A5SssC5wj4jjj5focHDm1D0k5hYsPYQ) | 2012 | to assess the level of depression among the cancer patients, to evaluate the effectiveness of counseling on depression among cancer patients and to compare the level of depression with their selected socio demographic variables. | Quasi - experimental study | ZSRDS | Our results indicate that the effects of counseling decrease the depression level. It is essential to raise the awareness and seek at-titudinal and behavioral changes among the health care professionals to tackle the psychological problems. |
| 1. Treatment of depression in cancer patients   [Treatment of depression in cancer patients - PubMed (nih.gov)](https://pubmed.ncbi.nlm.nih.gov/17938701/) | [G Rodin](https://pubmed.ncbi.nlm.nih.gov/?term=Rodin+G&cauthor_id=17938701), [M Katz](https://pubmed.ncbi.nlm.nih.gov/?term=Katz+M&cauthor_id=17938701), [N Lloyd](https://pubmed.ncbi.nlm.nih.gov/?term=Lloyd+N&cauthor_id=17938701), [E Green](https://pubmed.ncbi.nlm.nih.gov/?term=Green+E&cauthor_id=17938701), [J A Mackay](https://pubmed.ncbi.nlm.nih.gov/?term=Mackay+JA&cauthor_id=17938701), [R K S Wong](https://pubmed.ncbi.nlm.nih.gov/?term=Wong+RK&cauthor_id=17938701) | 2007 | Review of the evidence for the effectiveness of those therapies in patients with depression and cancer and developed the present clinical practice guideline based on that review and on expert consensus. | systematic review | Electronic data bases | The systematic review of the literature included eleven trials (seven of pharmacologic agents and four of non-pharmacologic interventions). Feedback received from 44 responding health care providers and the rap on the draft recommendations was addressed and documented in the guideline. Among providers, 82% agreed with the draft recommendations as stated, 68% agreed that the report should be approved as a practice guideline, and 73% indicated that they would be likely to use the guideline in their own practice. |
| 1. The Associations of Depression and Fatigue with Quality of Life among Palestinian Patients with Cancer   [The association of depression and fatigue with quality of life among Palestinian patients with cancer \| FADA ::Birzeit University Institutional Repository](https://fada.birzeit.edu/jspui/handle/20.500.11889/4111)  Google Scholar | [Dreidi, Mu'taz](https://fada.birzeit.edu/jspui/browse?type=author&value=Dreidi%2C+Mu%27taz) [Asmar, Imad](https://fada.birzeit.edu/jspui/browse?type=author&value=Asmar%2C+Imad) [Al-Rjoub, Belal](https://fada.birzeit.edu/jspui/browse?type=author&value=Al-Rjoub%2C+Belal) |  | To explore the relationships between depression, fatigue and QoL among patients diagnosed with cancer in the West Bank. | a descriptive, correlational study | Epidemiological Studies Depression Scale (CES-D) | Depression and fatigue were significantly correlated with QoL. The impact of depression should be taken into consideration by health care workers when designing or planning interventions to improve QoL for patients with cancer through accurate and early assessment. The moderate fatigue that patients with cancer experienced by the disease or by the treatment should also be taken into account. Each one of the key variables negatively affects QoL in separate. The occurrence of them simultaneously may increase the worse effect on QoL for patients with cancer. Based on that, depression and fatigue should be assessed for each patient with cancer. |
| Cancer care in the Palestinian territories [10.1016/S1470-2045(18)30323-1](http://dx.doi.org/10.1016/S1470-2045(18)30323-1)  Research gate | [Khalid Halahleh](https://www.researchgate.net/profile/Khalid-Halahleh-2?_sg%5B0%5D=85TD33iZo1_AHQI52-C9gmBbKhRcIHqEnEZNA6UnxSPy_RY8oTPHqRvh3dxzcU4exTZF--A.4MWp9ttBlHxMmAanWZzNABnzFZO2GRGSs34uGqazjWVbUuCo-iCOKtBAafFbSc5OUSPP4bPAG9zaXJp4q5VjxA&_sg%5B1%5D=PlNKGuwgxqfcwomEyHZ8fKdAhtWSyWwIQ_7CGGt3RQtP9WzFMPij_SlneAiJ1bwQWLokUSk.jZUXpDvYuQxSWM4ieOjGbepuob3xK2NeLkKb65RQftt56DhpseE6kDVat-D-pCtDFP-DhJ8hsFnjDvCKNWtfJg)  Robert Peter Gale | 2018 | Review depression treatments in Palestine | Review article | Electronic data bases | In this Review, we discuss the current state of cancer care in the Palestinian territories including epidemiology, screening, and prevention efforts, and infrastructural and workforce issues for the region. We also discuss examples of some encouraging progress that has been made for health in the region and the enormous challenges that the Palestinian health-care system still faces. |
| 1. Depressive symptoms and quality of life in home-care assisted patients   <http://dx.doi.org/10.1016/S0885-3924(96)00181-9>  Google Scholar | uigi Grassi, MD, Mania Indelli, MD, Marina Mazola, MD, Antonio Maestri, MD, Alessandra Santini, MD, Etick P&I, MD, and Massimo Boccalon, MD | 1996 | To examine the prevalence of depressive symptoms and its relationship with quality-of-life domains in home-care cancer patients at an advanced stage of illness | Quantitative | (Hospital Anxiety Depression Scale) (HAD) and quality of life (EORTC-QLQ-C30) | The quality of life of depressed patients was more affected than non-depressed patients in the social, emotional, cognitive, and physical domains. Significant correlations were found between depression scores and impairment in most quality-of-life areas. These findings support the importance of depression and quality-of-life evaluation in patients with advanced cancer who are followed in a home-care setting. This evaluation is needed to provide patients, their families, and caregivers with appropriate psychosocial interventions. |
| The impact of WeChat app-based education and rehabilitation program on anxiety, depression, quality of life, loss of follow-up and survival in non-small cell lung cancer patients who underwent surgical resection[The impact of WeChat app-based education and rehabilitation program on anxiety, depression, quality of life, loss of follow-up and survival in non-small cell lung cancer patients who underwent surgical resection - ScienceDirect](https://www.sciencedirect.com/science/article/abs/pii/S1462388919301759) Google Scholar | [YilingSui](https://www.sciencedirect.com/science/article/abs/pii/S1462388919301759#!)^[a](https://www.sciencedirect.com/science/article/abs/pii/S1462388919301759#!)^[TianWang](https://www.sciencedirect.com/science/article/abs/pii/S1462388919301759#!)^[a](https://www.sciencedirect.com/science/article/abs/pii/S1462388919301759#!)^[XiaochunWang](https://www.sciencedirect.com/science/article/abs/pii/S1462388919301759#!) | 2019 | to explore whether WeChat app-based education and rehabilitation program (WERP) affected anxiety, depression, quality of life (QoL), loss of follow-up and survival profiles in non-small cell lung cancer (NSCLC) patients after undergoing surgical resection. | a randomized, controlled study. | Hospital Anxiety and Depression Scale (HADS), and QoL was assessed by European Organization for Research and Treatment of Cancer Quality of Life Questionnaire (QLQ-C30). Loss of follow-up and survival data were also evaluated. | WERP is an effective way to improve well-being and quality of life in NSCLC patients after undergoing surgical resection. |
| Management of Depression in Patients with Cancer: A Clinical Practice Guideline[Management of Depression in Patients With Cancer: A Clinical Practice Guideline \| JCO Oncology Practice (ascopubs.org)](https://ascopubs.org/doi/pdf/10.1200/jop.2016.011072) Google Scholar | Madeline Li et al. | 2016 | This report updates the Cancer Care Ontario Program in Evidence-Based Care guideline for the management of depression in adult patients with cancer. This guideline covers pharmacologic, psychological, and collaborative care interventions, with a focus on integrating practical management tools to assist clinicians in delivering appropriate treatments for depression in patients with cancer. | A Clinical Practice Guideline (systematic review) | systematic review of Web sites of guideline developers, relevant cancer agencies, and MEDLINE and EMBASE | This updated guideline supports the previous general recommendation that patients with cancer who have depression may benefit from psychological and/or pharmacologic interventions, without evidence for the superiority of any specific treatment over another. New recommendations for a collaborative care model that incorporates a stepped care approach suggest that multidisciplinary mental health care restructuring may be required for optimal management of depression. |
| Prevalence of depression among cancer patients in Jordan: a national survey[Prevalence of depression among cancer patients in Jordan: a national survey - PubMed (nih.gov)](https://pubmed.ncbi.nlm.nih.gov/19242730/) | N. M. Mhaidat & K. H. Alzoubi & S. Al-Sweedan & B. A. Alhusein | 2009 | To investigate the prevalence of depression among cancer patients in Jordan | cross-sectional survey | Hospital Anxiety and Depression Scale (HADS) | The prevalence of depression in our sample was 51.9%.  In an effort to reduce the occurrence of depression among cancer patients, special attention is needed for changes in the psychological status in patients with knowledge about their diagnosis and patients in advanced disease stage. |
| Cancer-related post-treatment pain and its impact on health-related quality of life in breast cancer patients: a cross sectional study in Palestine[Cancer-related post-treatment pain and its impact on health-related quality of life in breast cancer patients: a cross sectional study in Palestine \| Asia Pacific Family Medicine \| Full Text (biomedcentral.com)](https://apfmj.biomedcentral.com/articles/10.1186/s12930-017-0037-3) PubMed | Nader H. Abu Farha , Mohammed T. Khatib , Husam Salameh and Sa’ed H. Zyoud | 2017 | To determine the QOL profile for breast cancer patients and stated the factors associated with QOL. | correlational cross-sectional study | Brief Pain Inventory (BPI)  (EQ-5D-5L) was used to examine HRQOL | Healthcare providers and health strategy makers should be alerted to low level HRQOL among breast cancer patients with low income level, patients with posttreatment pain, especially in the state of severe pain, and the state of pain interfering with daily life to improve their HRQOL.  Palliative care and social support for breast cancer patients improve the quality of life and reduce patient’s pain. |
| Assessment of Depression and Anxiety in Breast Cancer Patients Undergoing Radiotherapy in Palestine<https://doi.org/10.1007/s42399-020-00635-z> PubMed | Hussein ALMasri & Omar Rimawi | 2020 | s to assess experienced depression and anxiety among early breast cancer patients undergoing radiotherapy. | quantitative research method | e Hospital Anxiety and Depression Scale (HADS) | The highest anxiety and depression mean for treatment duration was for those treated for more than 3 months (SD = 3.2), and for those treated between 1 and 3 months (SD = 3), respectively. Some intervention may be needed to decrease the temporary anxiety and depression raised during radiotherapy for early-stage breast cancer patients. |
| The relationship between PTSD, Anxiety and Depression in Palestinian Children with Cancer and Mental Health of Mothers[The Relationship between PTSD, Anxiety and Depression in Palestinian Children with Cancer and Mental Health of Mothers (researchgate.net)](https://www.researchgate.net/profile/Abdel-Aziz/publication/318851468_The_Relationship_Between_Post-Traumatic_Stress_Disorder_and_Coping_Strategies_among_Patients_with_Cancer_in_Gaza_Strip/links/5a574b4645851547b1bfc2bd/The-Relationship-Between-Post-Traumatic-Stress-Disorder-and-Coping-Strategies-among-Patients-with-Cancer-in-Gaza-Strip.pdf) | Abdelaziz M Thabet and Mansour Mona A | 2017 | To investigate the prevalence of PTSD, depression and anxiety among children with cancer and relationship to mother’s mental health. | Mixed study | interview qƵĞƐƟŽnnĂŝrĞƐ  General Health questionnaire- 28 (GHQ-28 | The results from this study revealed that mental health among parents of children with cancer in WĂůĞƐƟnĞ is higher compared with their counterparts in the other contexts. The mental-health problems among mothers of children with cancer were higher than found in other studies [26-32]. There was no ĂƐƐŽcŝĂƟŽn between mother’s mental health and children PTSD, anxiety and depression and. Based on the results, paediatric oncology nurses can raise parents’ awareness about their mental-health problems, by ŝnƚĞrvĞnƟŽnƐ intended to decrease the risks. Parents could gain experience and ŝnĨŽrmĂƟŽn in group discussion, which provides appropriate opportunity for mothers to rĞŇĞcƚ on their own life stories. This life story ƉĞrƐƉĞcƟvĞ provides a rĞĂůŝƐƟc ĨŽƵnĚĂƟŽn that can support parents’ wellbeing and contribute to ƐĂƟƐĨy the needs through their children. |
| Herbal medicine for depression and anxiety: A systematic review with assessment of potential psycho-oncologic relevance[Herbal medicine for depression and anxiety: A systematic review with assessment of potential psycho-oncologic relevance - PubMed (nih.gov)](https://pubmed.ncbi.nlm.nih.gov/29464801/) | [K Simon Yeung](https://pubmed.ncbi.nlm.nih.gov/?term=Yeung+KS&cauthor_id=29464801) , [Marisol Hernandez](https://pubmed.ncbi.nlm.nih.gov/?term=Hernandez+M&cauthor_id=29464801) , [Jun J Mao](https://pubmed.ncbi.nlm.nih.gov/?term=Mao+JJ&cauthor_id=29464801) , [Ingrid Haviland](https://pubmed.ncbi.nlm.nih.gov/?term=Haviland+I&cauthor_id=29464801) , [Jyothirmai Gubili](https://pubmed.ncbi.nlm.nih.gov/?term=Gubili+J&cauthor_id=29464801) | 2018 | Identified single-herb medicines to treat anxiety and depression in cancer patients. | Systematic review | PubMed, Allied and Complementary Medicine, Embase, and Cochrane databases, selecting only single-herb randomized controlled trials between 1996 and 2016 in any population for data extraction | Overall, 45% of studies reported positive findings with fewer adverse effects compared with conventional medications. Based on available data, black cohosh, chamomile, chasteberry, lavender, passionflower, and saffron appear useful in mitigating anxiety or depression with favorable risk-benefit profiles compared to standard treatments. These may benefit cancer patients by minimizing medication load and accompanying side effects. However, well-designed larger clinical trials are needed before these herbs can be recommended and to further assess their psycho-oncologic relevance. |
| Depression and cancer<http://dx.doi.org/10.1590/S0101-60832009000900007> Google Scholar/SciELO | - [Sara Mota Borges Bottino](https://www.researchgate.net/profile/Sara-Bottino-3?_sg%5B0%5D=yHg-Ce3VvEBzLYIZ3AVrJr5hxSi8GnGoVT-cP4_ZPszVURuN_rRWFVDTNQQg9lLrCvlix4w.pDYb7z9RRo9qGbyrh31uuV6HPWlNtGoe6f5tx27wyLqGttbQz5urDVOsGB3VcB3M6aeFqoTSrmYSfimmeLYPwA&_sg%5B1%5D=qPVmqTgfZ-Q5xIvPQK2LhP1bBFQM383dGeL5xyI1bYpxnECU92LozPwCnp1wfUufOVyQVUM.-6hDkglwAAHBk9k_DaRErdVDY2iDYobjRhKeYH_cRtC0hiYn0tmIPqknNwIsfvxzyqTNpJJCZrVYED3ufeKzCw), - [Renerio Fraguas](https://www.researchgate.net/profile/Renerio_Fraguas?_sg%5B0%5D=yHg-Ce3VvEBzLYIZ3AVrJr5hxSi8GnGoVT-cP4_ZPszVURuN_rRWFVDTNQQg9lLrCvlix4w.pDYb7z9RRo9qGbyrh31uuV6HPWlNtGoe6f5tx27wyLqGttbQz5urDVOsGB3VcB3M6aeFqoTSrmYSfimmeLYPwA&_sg%5B1%5D=qPVmqTgfZ-Q5xIvPQK2LhP1bBFQM383dGeL5xyI1bYpxnECU92LozPwCnp1wfUufOVyQVUM.-6hDkglwAAHBk9k_DaRErdVDY2iDYobjRhKeYH_cRtC0hiYn0tmIPqknNwIsfvxzyqTNpJJCZrVYED3ufeKzCw) - ,[Wagner Gattaz](https://www.researchgate.net/profile/Wagner-Gattaz?_sg%5B0%5D=yHg-Ce3VvEBzLYIZ3AVrJr5hxSi8GnGoVT-cP4_ZPszVURuN_rRWFVDTNQQg9lLrCvlix4w.pDYb7z9RRo9qGbyrh31uuV6HPWlNtGoe6f5tx27wyLqGttbQz5urDVOsGB3VcB3M6aeFqoTSrmYSfimmeLYPwA&_sg%5B1%5D=qPVmqTgfZ-Q5xIvPQK2LhP1bBFQM383dGeL5xyI1bYpxnECU92LozPwCnp1wfUufOVyQVUM.-6hDkglwAAHBk9k_DaRErdVDY2iDYobjRhKeYH_cRtC0hiYn0tmIPqknNwIsfvxzyqTNpJJCZrVYED3ufeKzCw) | 2008 | To assist the assessment of depression in cancer patients and the indication for treatments pharmacological and psychosocial problems that have evidence in systematic reviews and good studies methodological quality. | Systematic review | Electronic data bases | Antidepressant treatments are effective, and improve the adherence to cancer treatments, reducing side effects as nausea, pain and fatigue. In cancer patients, pretreatment with antidepressants seems to minimize depressive symptoms induced by interferon-alfa. Psychosocial interventions as relaxing techniques, group and individual psychotherapies can also be applied to reduce depressive and stress symptoms in patients with cancer. |
| Religious beliefs, practices, and health in colorectal cancer patients in Saudi Arabia<https://doi.org/10.1002/pon.3845>Google Scholar | - Mahmoud Shaheen Al Ahwal , Faten Al Zaben, Mohammad Gamal Sehlo, Doaa Ahmed Khalifa and Harold G. Koenig | 2015 | examined the prevalence of religious beliefs and practices in colorectal cancer (CRC) patients and correlation with demographic, social, psychological, and physical health characteristics. | Seventy CRC patients (all Muslim) in Jeddah, Saudi Arabia | 13-item Muslim religiosity scale. | : All 70 participants (100%) engaged in group worship and prayer (Fard) five times/day, and 75.7% never skipped or combined two or more obligatory prayers; 71.4% read or recited the Qur’an several times/week or daily; 80.0% gave money to the poor each year (Zakat); 71.4% fasted throughout the month of Ramadan (Sawm) and other times as well; 91.4% said they ‘definitely’ experienced the presence of Allah; and 74.3% said their entire approach to life was definitely based on their religious beliefs. Overall religiosity was inversely related to depressive symptoms (B= 0.58, SE = 0.30, p = 0.026) and suicidal ideation (B= 0.07, SE = 0.03, p = 0.025), after controlling for financial status and social factors. Conclusions: Religious involvement was widespread in this sample of CRC patients in Saudi Arabia and was related to fewer depressive symptoms and less suicidal ideation. |
| Evaluation of Religious Coping in Tunisian Muslim Women with Newly Diagnosed Breast Cancer[Evaluation of Religious Coping in Tunisian Muslim Women with Newly Diagnosed Breast Cancer \| SpringerLink](https://link.springer.com/article/10.1007/s10943-020-01066-9)Google Scholar | - Feten Fekih‑Romdhane, Abir Hakiri1,Sana, FendriMehdi Balti, Raja Labbane,Majda Cheour | 2020 | evaluate religiosity and religious coping in a sample of breast cancer women, and to analyze the association between religiosity, religious coping, depression, anxiety, cancer clinical data, and sociodemographic data in our patients. | cross-sectional and descriptive study | The Depression Anxiety Stress Scales (DASS-21), the Arabic-Brief Religious Coping Scale (A-BRCS) and the Arabic Religiosity Scale. | High levels of affective religiosity were the main predictive factor of positive religious coping. Therapies should reinforce the positive religious coping patterns of breast cancer patients, and detect a possible resort to negative religious coping that may negatively affect the patients’ quality of life. |
| Social Support and Depression among Bone Marrow Transplant Patients<https://doi.org/10.1177%2F1359105307084310>Google Scholar | - JULIE D. JENKS KETTMANN & ELIZABETH M. ALTMAIER | 2008 | Considered the role of social support in mitigating depression among bone marrow transplant (BMT) patients. | Longitudinal study | Social Support Scale (MOSSSS) and the Centers for Epidemiological Studies of Depression Scale (CES-D) | Moderate levels of depressive symptoms in BMT patients, with 29.1 percent and 27.6 percent meeting the suggested criterion for clinical depression at pre-BMT and one year post-BMT, respectively. Overall, patients experienced a reduced level of depression post-BMT, although females reported more depression than males. Social support pre-BMT predicted depression levels post-BMT controlling for initial levels of depression. Clinical implications for health care providers working with cancer patients and their families are discussed. |
| Depression, Hopelessness and Social Support among Breast Cancer Patients: in Highly Endogamous Population<https://dx.doi.org/10.22034%2FAPJCP.2017.18.7.1889>Google Scholar | [Abdulbari Bener](https://www.ncbi.nlm.nih.gov/pubmed/?term=Bener%20A%5BAuthor%5D&cauthor=true&cauthor_uid=28749617), [Reem Alsulaiman](https://www.ncbi.nlm.nih.gov/pubmed/?term=Alsulaiman%20R%5BAuthor%5D&cauthor=true&cauthor_uid=28749617),[Lisa Doodson](https://www.ncbi.nlm.nih.gov/pubmed/?term=Doodson%20L%5BAuthor%5D&cauthor=true&cauthor_uid=28749617), and [Tony Agathangelou](https://www.ncbi.nlm.nih.gov/pubmed/?term=Agathangelou%20T%5BAuthor%5D&cauthor=true&cauthor_uid=28749617) |  | To assess the relationship between different demographic variables, hopelessness, depression and social support of Breast cancer patients in Qatari’s population. | Observational cohort hospital based study | Beck Hopelessness Scale (BHS), Back Depression Scale (BDS) and Multidimensional Scale of Perceived Social Support (MSPSS) | Hopelessness of the patients with breast cancer decreased with the increase in their social support. Therefore, activating patient social support systems is of importance in increasing their levels of hope. The present study revealed the coexistence of the socio-demographic, physical, psychological, and cognitive problems faced by patients with cancer. |
